# Alignment of Large Language Model Responses With Human Therapists in Motivational Interviewing

**DOI:** 10.1001/jamanetworkopen.2026.2750

**Published:** 2026-03-23

**Authors:** Bazen Gashaw Teferra, Sandra Huang, Nabil Johny, Argyrios Perivolaris, Huda Al-Shamali, Karisa Parkington, Alice Rueda, Richard J. Zeifman, Divya Sharma, Sri Krishnan, Candice Monson, Venkat Bhat

**Affiliations:** 1 Interventional Psychiatry Program, St Michael’s Hospital, Unity Health Toronto, Toronto, Ontario, Canada; 2 David R. Cheriton School of Computer Science, University of Waterloo, Waterloo, Ontario, Canada; 3 Faculty of Engineering, iBioMed Program, McMaster University, Hamilton, Ontario, Canada; 4 Department of Psychology, The New School for Social Research, New York, New York; 5 NYU Center for Psychedelic Medicine, NYU Grossman School of Medicine, New York, New York; 6 Centre for Psychedelic Research, Department of Brain Sciences, Faculty of Medicine, Imperial College London, London, United Kingdom; 7 Department of Mathematics and Statistics, York University, Toronto, Ontario, Canada; 8 Electrical, Computer, and Biomedical Engineering, Toronto Metropolitan University, Toronto, Ontario, Canada; 9 Department of Psychology, Toronto Metropolitan University, Toronto, Ontario, Canada; 10 Department of Psychiatry, University of Toronto, Toronto, Ontario, Canada

## Abstract

**Importance:**

Large language models (LLMs) are increasingly applied to mental health contexts, yet their capacity to generate responses that align with evidence-based psychotherapy remains uncertain. Motivational interviewing (MI), a structured counseling approach, provides an empirically grounded setting for evaluating alignment between LLM-generated and human therapist responses.

**Objective:**

To evaluate how closely an LLM’s responses align with therapist responses in MI sessions, using automated similarity metrics.

**Design, Setting, and Participants:**

This cross-sectional study used high-fidelity therapist-client transcripts annotated with the Motivational Interviewing Treatment Integrity system. Transcripts were sourced from publicly available counseling videos. For each therapist turn, the GPT-4o LLM generated a response using a standardized, MI-informed prompt based on the preceding conversation context. Analyses were conducted between March and May 2025.

**Main Outcomes and Measures:**

Alignment between LLM-generated and therapist responses was assessed using (1) cosine similarity based on sentence embeddings to capture semantic overlap and (2) DeepEval, a contextual deep-learning–based metric assessing coherence and contextual appropriateness. A therapist topic-consistency index quantified within-session thematic coherence and was examined as a moderator of alignment.

**Results:**

A total of 3706 therapist turns from 154 MI sessions were evaluated. Mean (SD) DeepEval scores were higher than mean (SD) cosine similarity scores (0.72 [0.31] vs 0.29 [0.20]; *P* < .001), suggesting limited semantic overlap despite greater contextual appropriateness. Therapist topic consistency significantly moderated similarity, where cosine similarity was higher in high-consistency than low-consistency sessions (mean [SD] difference, 0.027 [0.007]; *t*_3706_ = 3.987; *P* < .001), as was DeepEval score (mean [SD] difference, 0.038 [0.010]; *t*_3706_ = 3.747; *P* < .001). Correlation between metrics was negligible (Spearman ρ, –0.01), indicating that they captured distinct aspects of response alignment. LLM performance declined slightly across longer conversations (mean [SD] slope reduction for cosine similarity, −0.0005 [0.0016], and for DeepEval, −0.0005 [0.0022]), with increased verbosity and signs of reduced contextual grounding.

**Conclusions and Relevance:**

In this cross-sectional study of 154 MI sessions, prompted LLMs showed general alignment with therapist responses in MI-oriented conversations, as judged by automated similarity metrics. However, limitations in long-range coherence, stylistic alignment, and the use of indirect proxies for therapeutic quality highlight the need for improved prompt design, MI-specific evaluation methods, and clinical validation before integration into mental health care.

## Key Points

**Question:**

Can a large language model (LLM) generate therapist responses that align with human therapist turns in motivational interviewing (MI)–oriented conversations?

**Findings:**

In this cross-sectional study of 154 high-fidelity MI sessions (3706 therapist turns), the LLM showed low semantic similarity to therapist responses but higher contextual appropriateness. Alignment was significantly higher in sessions with greater therapist topic consistency and declined modestly over longer conversations.

**Meaning:**

The findings suggest LLMs can produce contextually appropriate MI-consistent responses, but limitations in coherence and stylistic alignment highlight the need for further validation before clinical use.

## Teaser

This cross-sectional study uses automated similarity metrics to examine how closely the responses of a large language model align with human therapist responses in motivational interviewing conversations.

## Introduction

The intersection of artificial intelligence (AI) and mental health care presents both remarkable opportunities and significant challenges.^1^ As mental health needs continue to outpace practitioner availability worldwide,^2^ AI-assisted therapeutic interventions have garnered increased attention.^3^ Large language models (LLMs) demonstrate sophisticated capabilities in natural language understanding^4^ and generation that could potentially support therapeutic interactions.^5^

Although recent research has explored LLMs’ application in mental health contexts,^6,7^ systematic evaluation of their ability to generate responses that align with structured, evidence-based psychotherapy encounters remains limited. This gap is critical because AI-generated responses that sound plausible but deviate from therapeutic principles can introduce ethical and clinical risks.^7^

Early conversational agents such as ELIZA^8^ mimicked therapeutic dialogue but lacked genuine understanding. Later systems grounded in cognitive behavioral therapy (CBT), such as Woebot^9^ and Wysa,^10^ were associated with improvements in subclinical anxiety and depression.^11^ Transformer-based LLMs^12^ further advanced these capabilities, enabling context-sensitive and coherent therapeutic interactions.^13,14^ Recent efforts have simulated both therapist and patient roles. For example, Wang et al^15^ developed PATIENT-ψ, a simulated patient framework based on CBT principles, and Yu and McGuinness^16^ integrated a fine-tuned DialoGPT model with therapeutic prompts to enhance emotional responsiveness.

In parallel, AI-based fidelity assessment platforms such as Lyssn.io^17^ have demonstrated that machine learning models can evaluate therapy quality and therapist empathy with expert-level reliability. Despite such advances, significant concerns persist about LLM reliability, safety, and adherence to therapeutic standards.^7,18^ These findings underscore the need for rigorous, domain-specific evaluation frameworks that ensure AI-generated dialogue reflects authentic therapeutic process.

Recent frameworks, including MHealth-EVAL,^19^ ψ-Arena,^20^ and others,^21^ have advanced context-aware, multiperspective evaluation of LLMs in mental health delivery.^19,20,21^ However, few studies explicitly test LLM adherence to structured therapeutic modalities, such as motivational interviewing (MI), across multiturn dialogues with human therapist benchmarks.

MI is a widely used communication style developed by Miller and Rollnick,^22^ supporting collaborative exploration of motivation for behavior change across clinical and health domains. Core MI skills, including open-ended questions, affirmations, reflections, and summaries (OARS), facilitate empathy, understanding, and client empowerment and are frequently used in substance use,^23^ medication adherence,^24^ chronic disease management,^25^ and broader behavioral and mental health settings. MI’s structured techniques can be reliably assessed (eg, through the Motivational Interviewing Treatment Integrity [MITI] system^26^), making MI-coded sessions a useful benchmark for comparing LLM and therapist responses.

This study introduced a framework for evaluating LLM alignment with therapist turns in MI-oriented dialogue. We assessed an LLM across annotated MI sessions, focusing on (1) LLM resemblance to therapist turns via automated similarity metrics, (2) performance evolution with increasing conversational context, and (3) moderation of alignment by therapist topic consistency. By combining semantic (cosine similarity) and contextual (DeepEval) metrics, this work clarifies LLM strengths and limitations in approximating therapist communication in MI-coded sessions

## Methods

### Dataset

This cross-sectional study used MI session transcripts between trained therapists and patients seeking behavior change, sourced from public YouTube channels and websites using an MI-specific search term. The dataset was introduced by Pérez-Rosas et al^27^ and has been used in prior computational counseling research, but it was not designed as a clinically validated corpus of routine therapeutic interactions. Therapist responses were drawn from MI-oriented counseling transcripts previously curated and evaluated using established MI frameworks, providing an expert-informed reference for alignment comparisons. Included videos featured educational demonstrations, supervised role-plays, or real counseling sessions addressing behavior change (eg, substance use, medication adherence, exercise, or diet). Only dyadic interactions (1 therapist, 1 client) aligned with MI principles were included. All analyses were cross-sectional, with similarity metrics computed once per therapist turn within a fixed corpus. All data were publicly available, with no identifiable participant information; therefore, institutional review board approval and informed consent were not required. This study followed the Transparent Reporting of a Multivariable Model for Individual Prognosis or Diagnosis (TRIPOD)-LLM reporting guideline,^28^ part of the EQUATOR Network family of checklists, to ensure transparency and reproducibility in the design, evaluation, and interpretation of LLM studies in health care contexts. It also followed the Strengthening the Reporting of Observational Studies in Epidemiology (STROBE) reporting guideline.

### Model Selection and Configuration

OpenAI’s GPT-4o (gpt-4o-mini-2024-07-18) was used, selected for its advanced reasoning capabilities. Model configurations were standardized across evaluation runs: temperature was set to 0.3, balancing creativity with consistency, and maximum response length was limited to 500 tokens to approximate therapist utterance. Rate limiting was implemented to comply with application programming interface usage policies. A uniform prompt structure was used across all turns, as described in the following section. Evaluations were conducted between March and May 2025 to ensure consistent model versions.

### Prompt Engineering

Prompt engineering was critical for aligning the LLM with MI principles and was refined through iterative testing. The final prompt included (1) role specification—explicit instruction for the LLM to assume the role of a therapist trained in MI; (2) MI principles—a brief overview of core MI principles (eg, empathy, discrepancy, resistance, and self-efficacy); (3) MI techniques—a description of specific techniques for conducting MI, including OARS; (4) conversation history—a chronologic presentation of all prior exchanges within the current session between the therapist and client; and (5) response instruction—a directive to generate the next therapist response using MI principles. Additionally, to enhance safety and factual reliability, a safety-check stage was incorporated in the prompt design, where responses were first scanned for self-harm, free-form personal data, or unjustified speculative content, with the system declining or redirecting such content.

Variations of this structure were systematically tested to optimize MI-consistent responses. The final prompt used to instruct the LLM to follow MI principles is given in eAppendix 1 in Supplement 1.

### System Architecture

To assess LLM adherence to MI principles, an evaluation framework was developed: (1) the context processor prepared the conversation history from the dataset for each evaluation point, formatting prior exchanges for input to the LLM; (2) the LLM response generator used engineered prompts to elicit therapist responses from the language model based on conversation context; (3) the evaluation compared LLM-generated responses with therapist responses using multiple evaluation methods; and (4) the analysis module aggregated results across conversation turns and generated visualizations. Each therapist’s turn was sequentially processed to preserve dialogue context and evaluate cumulative performance.

### Evaluation Metrics

Alignment between LLM-generated and therapist responses was assessed using 2 methods. First, we applied embedding similarity using SentenceTransformer’s all-MiniLM-L6-v2 model,^29^ due to its strong performance on semantic textual similarity tasks, to generate vector embeddings for LLM and therapist responses. Cosine similarity between these embeddings measured linguistic and semantic alignment, capturing meaning beyond surface-level text. The score ranges from −1 to 1, where a value of 1 indicates perfect word-by-word similarity, 0 indicates no similarity, and −1 indicates complete opposition.

Second, we used the DeepEval Assessment framework,^30^ which evaluates language models using prompt-based techniques. Unlike cosine similarity, which emphasizes surface-level or vector-space similarity, DeepEval is designed to capture higher-order dimensions of coherence, relevance, and alignment with conversational objectives. Based on deep learning methods, this produced a standardized similarity score assessing linguistic and contextual coherence, ranging from 0 to 1. A score of 0 represents failure to align, and a score of 1 indicates maximal alignment, where the generated response is deemed highly appropriate and contextually fitting. These metrics are intended as relative measures of response alignment under an identical conversational context rather than as absolute indicators of clinical quality. Additionally, cosine similarity and DeepEval scores are reported as descriptive benchmarks within the defined dataset; because the scales differ and no external comparator (eg, random or out-of-context responses) was included, these scores should not be interpreted as reaching or exceeding predefined clinical thresholds.

### Evaluation Procedure

For each therapist turn, the preceding conversation history was extracted, an LLM response was generated (using the standardized prompt), and the corresponding human therapist response was retrieved. We then compared the 2 responses using cosine similarity and DeepEval scoring. All outputs, scores, and metadata were recorded for analysis.

Aggregate statistics were computed after processing all sessions, and visualizations were generated to illustrate metric trends. Representative examples supported qualitative interpretation.

The pipeline was implemented in custom Python software with integrated logging and error handling. An overview of the evaluation workflow is presented in eAppendix 2 in Supplement 1.

### Categories Based on Topic Consistency

Therapist topic consistency was quantified by computing the mean cosine similarity between consecutive therapist utterances within each transcript using the all-MiniLM-L6-v2 model.^29^ Sessions were split at the median into high- and low-consistency groups to examine moderation of LLM alignment.

We note that this binary dichotomization simplifies the underlying continuum of coherence. As such, this grouping served as a general moderator analysis, and we recognize that future work should explore multilevel or continuous modeling of therapist consistency.

### Statistical Analysis

Analyses were conducted in Python, version 3.13 (Python Software Foundation), using NumPy, SciPy, and Statsmodels packages. We computed descriptive statistics for each evaluation metric. Correlation analyses examined correlations between cosine similarity and DeepEval scores. Independent 2-tailed *t* tests and Mann-Whitney *U* tests (α = .05) compared high- and low-consistency groups. Two-sided *P* < .05 was considered significant. The custom Python software developed for this study (implementation, metric computation, high- or low-consistency group assignment, statistical analyses, and result visualizations) is open-source and publicly available.^31^

## Results

### Aggregate Similarity Performance

From 259 identified sessions, transcripts were automatically captioned and human-verified. Following MITI-based quality review,^26^ 154 high-fidelity MI sessions (3706 therapist turns) met the inclusion criteria. To measure alignment between LLM-generated and therapist responses, we calculated cosine similarity and DeepEval scores across all therapist turns.

**Figure 1.**
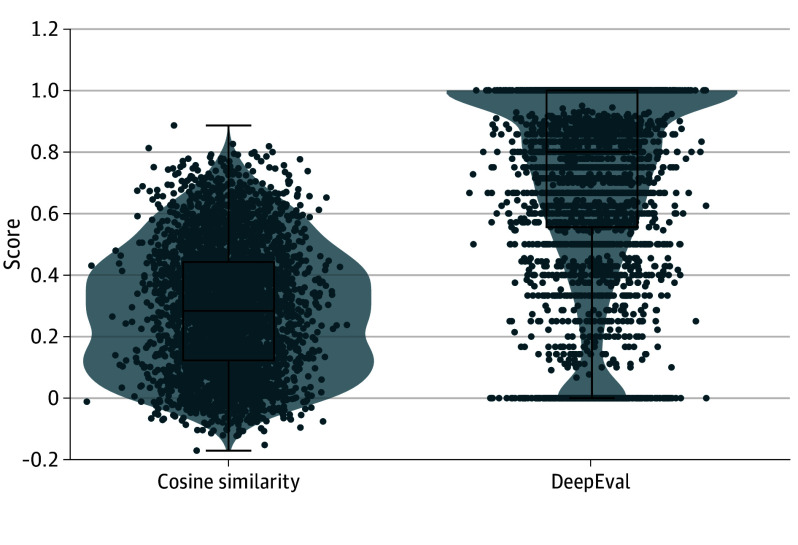
Box Plots and Violin Plots Illustrating the Distribution of Cosine Similarity and DeepEval Contextual Evaluation Scores Across All 3706 Therapist–Large Language Model (LLM) Comparison Turns The horizontal bar inside the boxes indicates the median and the lower and upper ends of the boxes, the first and third quartiles. The whiskers indicate SDs. Cosine similarity values reflect lexical and semantic overlap between LLM and therapist responses (score range, −1 to 1, with 1 indicating perfect word-by-word similarity; 0, no similarity; and −1, complete opposition). DeepEval scores indicate whether LLM responses were contextually appropriate despite differences in wording (score range, 0-1, with 0 representing no alignment and 1, maximal alignment).

Mean (SD) DeepEval scores were higher than mean (SD) cosine similarity scores (0.72 [0.31] vs 0.29 [0.20]; *P* < .001), suggesting limited semantic overlap despite greater contextual appropriateness. DeepEval scores showed a higher central tendency and broader dispersion within their own scale, whereas cosine similarity values were more narrowly distributed, reflecting the distinct constructs captured by the 2 metrics (Table 1 and Figure 1). eAppendix 3 in Supplement 1 presents actual examples from the dataset to be used as a benchmark with a cosine similarity and DeepEval score that are close to the mean values in Table 1.

**Table 1.** Descriptive Statistics for Similarity Metrics of the Large Language Model vs a Human Therapist

Metric	Mean (SD)	Median (range)
Cosine similarity	0.29 (0.20)	0.28 (−0.17 to 0.89)
DeepEval score	0.72 (0.31)	0.80 (0.00 to 1.00)

Figure 1 presents a comparative box plot of both metrics, with error bars showing SDs. The figure also presents violin plots to illustrate the full distribution, emphasizing the bimodal nature of DeepEval and the left-skewed nature of cosine similarity.

### Therapist Consistency and Similarity Scores

We next examined whether the consistency of human therapists was associated with LLM alignment. Results are presented in Table 2.

**Table 2.** Descriptive Statistics for Cosine Similarity and DeepEval Scores by Consistency Group

Metric and group	Mean (SD)	Median (range)
Cosine similarity		
High consistency	0.31 (0.21)	0.30 (−0.12 to 0.89)
Low consistency	0.28 (0.20)	0.27 (−0.17 to 0.80)
DeepEval score		
High consistency	0.74 (0.30)	0.83 (0.00 to 1.00)
Low consistency	0.70 (0.32)	0.80 (0.00 to 1.00)

Cosine similarity was higher in high-consistency than low-consistency sessions (mean [SD] difference, 0.027 [0.007]; *t*_3706_ = 3.987; *P* < .001), as was DeepEval score (mean [SD] difference, 0.038 [0.010]; *t*_3706_ = 3.747; *P* < .001). Mann-Whitney *U* tests produced similar results (cosine similarity: *U* = 1 786 043 [*P* < .001]; DeepEval: *U* = 1 766 911 [*P* = .001]).

**Figure 2.**
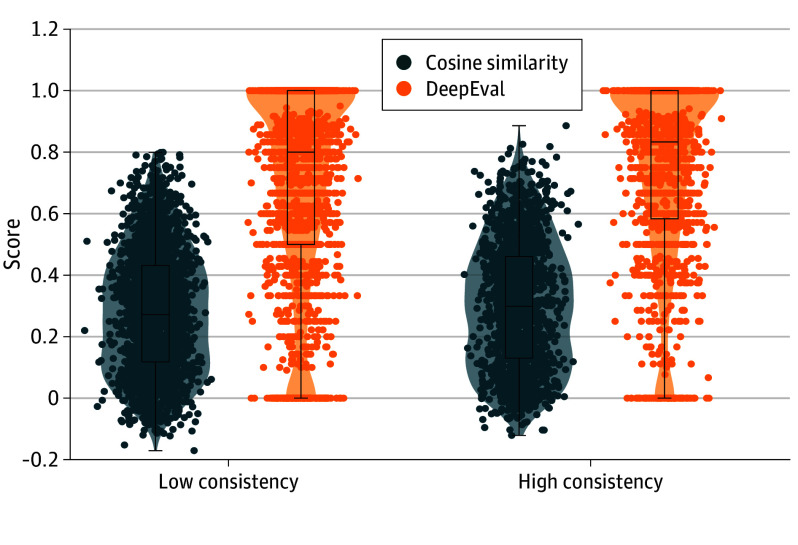
Box Plots and Violin Plots Comparing Cosine Similarity and DeepEval Contextual Evaluation Scores for Sessions Categorized by Therapist Topic Consistency The horizontal bar inside the boxes indicates the median and the lower and upper ends of the boxes, the first and third quartiles. The whiskers indicate SDs. Therapist topic consistency was calculated as the mean cosine similarity between consecutive therapist utterances within each session, and transcripts were split at the median into low- and high-consistency groups. Cosine similarity values reflect lexical and semantic overlap between LLM and therapist responses (score range, −1 to 1, with 1 indicating perfect word-by-word similarity; 0, no similarity; and −1, complete opposition). DeepEval scores indicate whether LLM responses were contextually appropriate despite differences in wording (score range, 0-1, with 0 representing no alignment and 1, maximal alignment).

These results suggest that therapist consistency may significantly moderate the extent to which LLMs can emulate therapeutic communication. Specifically, LLM alignment scores were higher when therapists were more consistent, as shown in Figure 2, indicating that LLMs aligned more closely with therapists whose responses exhibited greater internal consistency.

### Metric Comparison and Agreement Analysis

To assess agreement between evaluation metrics, we computed Spearman rank order correlation coefficients between cosine similarity and the DeepEval score for all turns. Results showed that the correlation was negligible, indicating minimal monotonic association between the 2 metrics (Spearman ρ, −0.01; *P* = .64).

For visualization purposes, we present a quadrant plot in eAppendix 4 in Supplement 1 to illustrate the divergence between the 2 metrics, categorizing response pairs into 4 groups. The thresholds were selected by taking the mean value (as presented in Table 1) for both the cosine similarity metric and the DeepEval score (both high: cosine >0.29, DeepEval ≥0.72; both low: cosine ≤0.29, DeepEval <0.72; cosine high, DeepEval low: cosine >0.29, DeepEval <0.72; cosine low, DeepEval high: cosine ≤0.29, DeepEval ≥0.72).

### Temporal Dynamics in Conversations

To analyze performance trends, the slopes of similarity scores as the conversation progressed through time were calculated, and histograms across all sessions were generated. The histogram plot is presented in eAppendix 5 in Supplement 1. Slopes were used as a parsimonious summary of within-session temporal trends. However, individual session trajectories may exhibit heterogeneity that is not fully captured by a single linear estimate. The plots show that the slopes of the similarity score ranged from negative to positive, but the mean was slightly negative (mean [SD] slope reduction for cosine similarity, −0.0005 [0.0016], and for DeepEval, −0.0005 [0.0022]). These suggest that the similarity score for cosine similarity and DeepEval decreased, on average, as a conversation continued. This indicates LLMs may struggle with long-range coherence or accumulate topic drift. Table 3 summarizes the distribution of the slopes of the similarity score as conversations progressed.

**Table 3.** Descriptive Statistics for the Slopes of the Similarity Score

Metric	MI sessions, No.	Mean (SD) [95% CI]	Median (IQR)
All cosine similarity scores	154	−0.0005 (0.0016) [−0.00075 to −0.00023]	−0.0005 (−0.0014 to 0.0003)
All DeepEval scores	154	−0.0005 (0.0022) [−0.00084 to −0.00015]	−0.0006 (−0.0018 to 0.0008)

Abbreviation: MI, motivational interviewing.

### Word Count Disparities

LLM responses were substantially longer than therapist utterances (eAppendix 6 in Supplement 1). This verbosity may have influenced both evaluation metrics. The inflated word count suggests a stylistic mismatch that may affect perceived therapeutic fidelity and efficiency.

### Error Case Analysis

Analysis of low-similarity cases (eAppendix 7 in Supplement 1) revealed systematic differences in conversational strategy. Therapists more often prioritized procedural tasks such as clarification or session closure, whereas the LLM emphasized engagement, reflection, and conversational continuity. Therapists tended to pace sessions efficiently and ask direct follow-up questions, while the LLM frequently used empathic statements and reflective listening before progressing.

## Discussion

### Evaluation Metric Divergence

Cosine similarity and DeepEval scores revealed distinct perspectives on therapist-LLM alignment. Their weak correlation (Spearman ρ, −0.01) suggests they captured different aspects of response quality, with cosine similarity emphasizing linguistic overlap and DeepEval capturing intent and therapeutic appropriateness. Accordingly, the cosine similarity of 0.29 represented modest semantic overlap typical of distinct yet contextually related human responses, while higher DeepEval scores indicated contextual appropriateness rather than stylistic equivalence. This highlights the need for multidimensional, task-specific frameworks rather than single semantic metrics.

### Therapist Consistency as a Moderating Factor

Performance was higher in sessions with thematically consistent therapists, suggesting that LLMs mirror the clarity and structure of their input prompts. Prior work similarly showed that topic coherence guides model relevance.^32^

In psychotherapy, such consistency supports engagement and alliance.^33^ Training on coherent, high-fidelity dialogue may improve realism and therapeutic fidelity. However, declining performance in broader discussions underscores limitations in long-context reasoning and the need for training data that preserve contextual continuity, particularly in clinically sensitive conversations where incoherence could erode trust.

We derived the high- and low-consistency groups by splitting therapist topic consistency scores at the median. While this binary split enabled a clear comparison between groups, we recognize that it reduced the granularity of consistency variation. Future work should explore multilevel consistency bins or continuous modeling of therapist coherence to capture more nuanced effects.

### Longitudinal Degradation and Contextual Challenges

Similarity scores declined as sessions progressed, potentially reflecting topic drift, memory limitations, or increasing complexity. Prior work showed that LLMs often struggle with long-context reasoning, leading to generic or misaligned responses in later turns.^34^ While early-session LLM responses in the current study often matched therapists in tone and direction, later turns showed divergence, generic phrasing, or missed opportunities for deepening the conversation. These issues reflect broader limitations in long-context reasoning and point to practical constraints for the deployment of LLMs in extended therapeutic dialogues.

### Stylistic Mismatch and Verbosity

LLM responses were longer and more verbose than therapist responses, which may inflate evaluation scores but reduce fidelity. This mirrors prior findings in LLMs that verbosity may be mistaken for depth.^35^ From a deployment perspective, concise, focused responses may better match clinical dialogue norms and improve clinician acceptance.

### Error Cases

Analysis of cases with low similarity found systematic differences in conversational strategy, with therapists more often prioritizing procedural tasks, session pacing, and direct follow-up questions and the LLM emphasizing engagement, reflection, conversational continuity, empathic statements, and reflective listening. Although these LLM tendencies can align well with MI principles, they may also slow session flow or diverge from immediate therapeutic goals. These contrasts highlight complementary strengths and limitations, suggesting opportunities to refine LLM prompting to better balance MI-consistent empathy with task-focused clinical efficiency.

### Drawbacks of Current Approaches

LLMs often produce reflective yet vague responses, underscoring the need for targeted fine-tuning and stricter prompt constraints. While DeepEval provides a nuanced assessment, it remains a black-box metric with possible bias. Current models also lack personalization to client history or goals, a core feature of psychotherapy. Future work should integrate human assessments and MI-specific coding systems (eg, MITI). Similarly, following Richard Sutton’s “bitter lesson,”^36^ it is possible that LLMs could ultimately achieve more effective therapeutic interventions not by strictly adhering to human-designed fidelity frameworks such as MI or MITI but by learning directly from data, suggesting that rule-based adherence may limit their long-term potential.

### Future Directions for LLMs and Psychotherapy

This study highlights both the promise and limitations of using LLMs in psychotherapy. While capable of generating therapeutically appropriate responses, current models struggle with clinical specificity, coherence, and intent alignment.^37^ Misaligned AI responses could erode trust or reinforce maladaptive narratives, particularly in vulnerable populations.

Future work could improve evaluation procedures by introducing out-of-context controls, comparing multiple human therapist benchmarks, and incorporating expert ratings to contextualize automated metrics. Although we selected our LLM to represent a contemporary high-capacity LLM, its proprietary nature limits interpretability; studies using open-weight models may clarify how architecture and training data influence therapeutic alignment.

Safe integration of LLMs into mental health care will require human oversight, transparency, and defined boundaries of use. Domain-specific fine-tuning, real-time MI coding, hybrid semantic-clinical metrics, and inclusion of clinician and patient perspectives will be essential for ethical and effective clinical translation.

### Limitations

Several limitations of this study warrant consideration. The dataset size and single-session format limit representativeness across therapist styles, and the publicly available transcripts were not derived from a clinically validated therapy corpus. Future work should validate findings on prospectively collected clinical datasets. Automated metrics, while objective, may not capture nuanced clinical judgment, and evaluation was limited to a single LLM using a fixed prompt, restricting generalizability. Analyses were conducted at the turn level without explicitly modeling within-session correlation. Future work should use hierarchical or mixed-effects approaches to disentangle within- and between-session variability in alignment metrics. In addition, MI’s structured nature may limit generalizability to less formalized psychotherapy modalities.

## Conclusions

In this cross-sectional study of 154 MI sessions, a framework to evaluate how LLMs align with therapist responses in MI-coded simulated therapeutic dialogue was developed. Using cosine similarity and DeepEval metrics, we found that performance varied by evaluation type, therapist consistency, and conversation stage. The findings suggest that LLMs can approximate MI-consistent therapist responses but often struggle with clinical grounding and long-context sensitivity. The influence of therapist consistency underscores the need for high-quality training data and improved prompting methods. This study’s findings demonstrate general alignment within the evaluated dataset but do not establish clinical equivalence. Future work should refine evaluation methods to better capture therapeutic intent and align LLM development with specific clinical applications. Integrating automated metrics with human evaluation and enhancing long-range coherence modeling will be essential to ensure ethical and effective AI use in psychotherapy.

## Notes

Supplement 1. **eAppendix 1.** Prompt to Instruct LLM to Follow Motivational Interviewing Principles **eAppendix 2.** Evaluation Procedure **eAppendix 3.** Benchmarking the Mean Cosine Similarity and DeepEval Scores **eAppendix 4.** Quadrant Plot **eAppendix 5.** Temporal Dynamics in Conversations **eAppendix 6.** Word Count Summary **eAppendix 7.** Error Cases Examples

Supplement 2.
Data Sharing Statement

## References

[1] Olawade DB, Wada OZ, Odetayo A, David-Olawade AC, Asaolu F, Eberhardt J. Enhancing mental health with artificial intelligence: current trends and future prospects. J Med Surg Public Health. 2024;3:100099. doi:10.1016/j.glmedi.2024.100099

[2] Phillips L. A closer look at the mental health provider shortage. *Counseling Today*. May 2023. Accessed June 23, 2025. https://www.counseling.org/publications/counseling-today-magazine/article-archive/article/legacy/a-closer-look-at-the-mental-health-provider-shortage

[3] Hua Y, Na H, Li Z, . A scoping review of large language models for generative tasks in mental health care. NPJ Digit Med. 2025;8(1):230. doi:10.1038/s41746-025-01611-4 40307331 PMC12043943

[4] Teferra BG, Rueda A, Pang H, . Screening for depression using natural language processing: literature review. Interact J Med Res. 2024;13:e55067. doi:10.2196/55067 39496145 PMC11574504

[5] Thakkar A, Gupta A, De Sousa A. Artificial intelligence in positive mental health: a narrative review. Front Digit Health. 2024;6:1280235. doi:10.3389/fdgth.2024.1280235 38562663 PMC10982476

[6] Teferra BG, Rose J. Predicting generalized anxiety disorder from impromptu speech transcripts using context-aware transformer-based neural networks: model evaluation study. JMIR Ment Health. 2023;10:e44325. doi:10.2196/44325 36976636 PMC10131846

[7] Guo Z, Lai A, Thygesen JH, Farrington J, Keen T, Li K. Large language models for mental health applications: systematic review. JMIR Ment Health. 2024;11:e57400. doi:10.2196/57400 39423368 PMC11530718

[8] Weizenbaum J. ELIZA—a computer program for the study of natural language communication between man and machine. Commun ACM. 1966;9(1):36-45. doi:10.1145/365153.365168

[9] Fitzpatrick KK, Darcy A, Vierhile M. Delivering cognitive behavior therapy to young adults with symptoms of depression and anxiety using a fully automated conversational agent (Woebot): a randomized controlled trial. JMIR Ment Health. 2017;4(2):e19. doi:10.2196/mental.7785 28588005 PMC5478797

[10] Gupta M, Malik T, Sinha C. Delivery of a mental health intervention for chronic pain through an artificial intelligence-enabled app (Wysa): protocol for a prospective pilot study. JMIR Res Protoc. 2022;11(3):e36910. doi:10.2196/36910 35314423 PMC9015778

[11] Li H, Zhang R, Lee YC, Kraut RE, Mohr DC. Systematic review and meta-analysis of AI-based conversational agents for promoting mental health and well-being. NPJ Digit Med. 2023;6(1):236. doi:10.1038/s41746-023-00979-5 38114588 PMC10730549

[12] Vaswani A, Shazeer N, Parmar N, . Attention is all you need. *arXiv*. Preprint posted online December 5, 2017. doi:10.48550/arXiv.1706.03762

[13] Nerella S, Bandyopadhyay S, Zhang J, . Transformers and large language models in healthcare: a review. Artif Intell Med. 2024;154:102900. doi:10.1016/j.artmed.2024.102900 38878555 PMC11638972

[14] Greco CM, Simeri A, Tagarelli A, Zumpano E. Transformer-based language models for mental health issues: a survey. Pattern Recognit Lett. 2023;167:204-211. doi:10.1016/j.patrec.2023.02.016

[15] Wang R, Milani S, Chiu JC, . PATIENT-ψ: using large language models to simulate patients for training mental health professionals. In: *Proceedings of the 2024 Conference on Empirical Methods in Natural Language Processing*. Association for Computational Linguistics; 2024:12772-12797.

[16] Yu HQ, McGuinness S. An experimental study of integrating fine-tuned large language models and prompts for enhancing mental health support chatbot system. J Med Artif Intell. 2024;7:16. doi:10.21037/jmai-23-136

[17] Creed TA, Salama L, Slevin R, . Enhancing the quality of cognitive behavioral therapy in community mental health through artificial intelligence generated fidelity feedback (Project AFFECT): a study protocol. BMC Health Serv Res. 2022;22(1):1177. doi:10.1186/s12913-022-08519-9 36127689 PMC9487132

[18] Dergaa I, Fekih-Romdhane F, Hallit S, . ChatGPT is not ready yet for use in providing mental health assessment and interventions. Front Psychiatry. 2024;14:1277756. doi:10.3389/fpsyt.2023.1277756 38239905 PMC10794665

[19] Chen L, Preece DA, Sikka P, Gross JJ, Krause B. A framework for evaluating appropriateness, trustworthiness, and safety in mental wellness AI chatbots. *arXiv*. Preprint posted online July 16, 2024. doi:10.48550/arXiv.2407.11387

[20] Zhu S, Chen Z, Bi G, . ψ-Arena: interactive assessment and optimization of LLM-based psychological counselors with tripartite feedback. *arXiv*. Preprint posted online May 6, 2025. doi:10.48550/arXiv.2505.03293

[21] Marrapese A, Suleiman B, Ullah I, Kim J. A novel nuanced conversation evaluation framework for large language models in mental health. *arXiv*. Preprint posted online March 8, 2024. doi:10.48550/arXiv.2403.09705

[22] Miller WR, Rollnick S. Motivational Interviewing: Helping People Change. Guilford Press; 2012.

[23] Schwenker R, Dietrich CE, Hirpa S, . Motivational interviewing for substance use reduction. Cochrane Database Syst Rev. 2023;12(12):CD008063. 38084817 10.1002/14651858.CD008063.pub3 PMC10714668

[24] Palacio A, Garay D, Langer B, Taylor J, Wood BA, Tamariz L. Motivational interviewing improves medication adherence: a systematic review and meta-analysis. J Gen Intern Med. 2016;31(8):929-940. doi:10.1007/s11606-016-3685-3 27160414 PMC4945560

[25] Coyne N, Correnti D. Effectiveness of motivational interviewing to improve chronic condition self-management: what does the research show us? Home Healthc Nurse. 2014;32(1):56-63. doi:10.1097/NHH.0000000000000001 24326477

[26] Moyers TB, Rowell LN, Manuel JK, Ernst D, Houck JM. The Motivational Interviewing Treatment Integrity Code (MITI 4): rationale, preliminary reliability, and validity. J Subst Abuse Treat. 2016;65:36-42. doi:10.1016/j.jsat.2016.01.001 26874558 PMC5539964

[27] Pérez-Rosas V, Wu X, Resnicow K, Mihalcea R. What makes a good counselor? learning to distinguish between high-quality and low-quality counseling conversations. In: *Proceedings of the 57th Annual Meeting of the Association for Computational Linguistics*. Association for Computational Linguistics; 2019:926-935.

[28] Gallifant J, Afshar M, Ameen S, . The TRIPOD-LLM reporting guideline for studies using large language models. Nat Med. 2025;31(1):60-69. doi:10.1038/s41591-024-03425-5 39779929 PMC12104976

[29] Galli C, Donos N, Calciolari E. Performance of 4 pre-trained sentence transformer models in the semantic query of a systematic review dataset on peri-implantitis. Information (Basel). 2024;15(2):68. doi:10.3390/info15020068

[30] Li J, Li R, Liu Q. Beyond static datasets: a deep interaction approach to LLM evaluation. *arXiv*. Preprint posted online September 8, 2023. doi:10.48550/arXiv.2309.04369

[31] *TherapySimulation*. Version 1.0. July 7, 2025. Accessed August 4, 2025. https://github.com/teferrabg/TherapySimulation.git

[32] Rahimi H, Mimno D, Hoover J, Naacke H, Constantin C, Amann B. Contextualized topic coherence metrics. In: Graham Y, Purver M, eds. *Findings of the Association for Computational Linguistics: EACL 2024*. Association for Computational Linguistics; 2024:1760-1773. Accessed April 9, 2025. https://aclanthology.org/2024.findings-eacl.123/

[33] Stubbe DE. The therapeutic alliance: the fundamental element of psychotherapy. Focus (Am Psychiatr Publ). 2018;16(4):402-403. doi:10.1176/appi.focus.20180022 31975934 PMC6493237

[34] Kuratov Y, Bulatov A, Anokhin P, . BABILong: testing the limits of LLMs with long context reasoning-in-a-haystack. *arXiv*. Preprint posted online June 14, 2024. doi:10.48550/arXiv.2406.10149

[35] Zhang Y, Das SSS, Zhang R. Verbosity ≠ veracity: demystify verbosity compensation behavior of large language models. *arXiv*. Preprint posted online December 7, 2024. doi:10.48550/arXiv.2411.07858

[36] Sutton R. The bitter lesson of machine learning. KDnuggets. Accessed September 4, 2025. https://www.kdnuggets.com/the-bitter-lesson-of-machine-learning

[37] Na H, Hua Y, Wang Z, . 2025. A survey of large language models in psychotherapy: current landscape and future directions. In: Che W, Nabende J, Shutova E, Pilehvar MT, eds. *Findings of the Association for Computational Linguistics: ACL 2025*. Association for Computational Linguistics; 2025:7362-7376. Accessed February 13, 2026. https://aclanthology.org/2025.findings-acl.385/

