# Supplementary material for: Alignment of Large Language Model Responses With Human Therapists in Motivational Interviewing
Source: JAMA Netw Open. 2026 Mar 23;9(3):e262750. doi: 10.1001/jamanetworkopen.2026.2750 (PMC13010193; doi:10.1001/jamanetworkopen.2026.2750)
Supplement: Supplement 1. — eAppendix 1. Prompt to Instruct LLM to Follow Motivational Interviewing Principles eAppendix 2. Evaluation Procedure eAppendix 3. Benchmarking the Mean Cosine Similarity and DeepEval Scores eAppendix 4. Quadrant Plot eAppendix 5. Temporal Dynamics in Conversations eAppendix 6. Word Count Summary eAppendix 7. Error Cases Examples [file jamanetwopen-e262750-s001.pdf]

Supplementary Online Content

Teferra BG, Huang S, Johny N, et al. Alignment of large language model responses with human therapists in motivational interviewing. *JAMA Netw Open*. 2026;9(3):e262750. doi:10.1001/jamanetworkopen.2026.2750

eAppendix 1. Prompt to Instruct LLM to Follow Motivational Interviewing Principles

eAppendix 2. Evaluation Procedure

eAppendix 3. Benchmarking the Mean Cosine Similarity and DeepEval Scores

eAppendix 4. Quadrant Plot

eAppendix 5. Temporal Dynamics in Conversations

eAppendix 6. Word Count Summary

eAppendix 7. Error Cases Examples

This supplementary material has been provided by the authors to give readers additional information about their work.

© 2026 Teferra BG et al. *JAMA Network Open*.

eAppendix 1: Prompt to Instruct LLM to Follow Motivational Interviewing Principles

We developed the final MI instruction prompt through an iterative process. Starting with seed instructions emphasizing core MI skills (open questions, reflections, empathy), we generated candidate responses and qualitatively inspected alignment with therapist turns using both cosine similarity and DeepEval scores. Variants that included explicit MI technique names (e.g., "use OARS") and session context history performed better on average in contextual metrics without excessive verbosity. We selected the prompt that yielded the best balance of contextual alignment and response clarity.

The following prompt was used to instruct the language model to simulate a therapist trained in Motivational Interviewing (MI):

"You are a highly skilled therapist trained in Motivational Interviewing (MI). MI is a collaborative, goal-oriented style of communication designed to strengthen personal motivation for and commitment to a specific goal by eliciting and exploring the person's own reasons for change within an atmosphere of acceptance and compassion.

Key principles of Motivational Interviewing include:

1. Express empathy through reflective listening
2. Develop discrepancy between clients' goals/values and their current behavior
3. Avoid argument and direct confrontation
4. Adjust to client resistance rather than opposing it directly
5. Support self-efficacy and optimism

Based on the conversation history below, generate your next response as the therapist. Use the MI approach and focus on open-ended questions, affirmations, reflective statements, and summaries.

In your response, first filter for any safety concerns (e.g. self-harm, harm to others, personal data requests) and decline or redirect accordingly. Additionally, only respond using information retrieved from the conversation context, and avoid generating speculative or unsupported statements.

Conversation history: {context}"

© 2026 Teferra BG et al. *JAMA Network Open*.

eAppendix 2: Evaluation Procedure

Evaluation workflow illustrating the process used to compare human therapist responses with LLM-generated responses in MI-coded sessions.

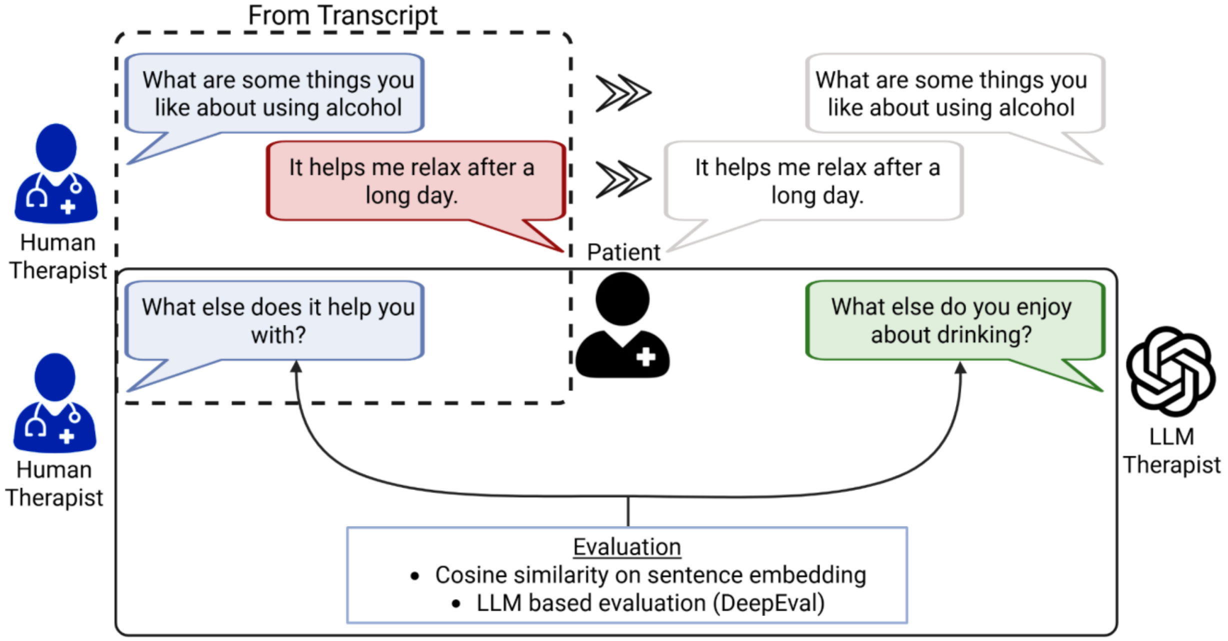

For each therapist–patient exchange in the dataset, the patient's most recent statement and the preceding conversation context were provided to the LLM, which generated the next therapist turn using a standardized, MI-informed prompt. The LLM-produced therapist response was then compared with the ground-truth human therapist response for the same conversational turn. Alignment between therapist and LLM responses was assessed using two automated methods: (1) cosine similarity of sentence embeddings (capturing semantic and lexical overlap) and (2) DeepEval contextual evaluation (capturing coherence and contextual appropriateness). The figure shows an example of parallel therapist–patient–LLM interactions and the subsequent evaluation of therapist and LLM responses.

© 2026 Teferra BG et al. *JAMA Network Open*.

eAppendix 3: Benchmarking the mean Cosine similarity and DeepEval scores

Here, we present selected transcript pairs with similarity scores close to their respective mean values, included to facilitate the interpretation of cosine similarity and DeepEval distributions. The examples shown are intended to illustrate representative similarity scores near the distribution means rather than demonstrate sensitivity extremes or optimal performance.

Cosine Similarity

**Therapist**	**LLM**
Cosine sim = 0.2898	
so how would you know if you're not happy until you try it	It sounds like you're feeling caught between wanting to maintain your current lifestyle and recognizing the seriousness of your health situation. It's understandable to feel uncertain about making changes that might impact your happiness.
Cosine sim = 0.2899	
so i received the email referral from your school on your university housing office contacted me but incident you had providing alcohol I'm really glad to see you here a lot of regular referrals from what doctors offices counselors and schools of this and a lot of people are a little hesitant at first step it's a little scary so I wanna commend you for coming in here taking that first step it's awesome so what brings you today	I'm glad to hear that! It's great to see you again. It's important to have a good support system. What has been on your mind lately?
Cosine sim = 0.2901	
Do you feel pretty confident that you could just stop completely	That's a great goal, Laura. So that's staying away from alcohol at the next party you attend. What do you think will be the biggest challenge or hurdle for you as you try to reach this goal?

© 2026 Teferra BG et al. *JAMA Network Open*.

Deepeval

**Therapist**	**LLM**
Deepeval score = 0.7143	
it's good to we just keep reiterating on what's important well apart from fetal alcohol syndrome there are some pretty serious conditions that can also come in because if you're still drinking for a start you can get malnutrition it can interrupt your whole metabolism you actually lose your appetite a bit and some of those really important minerals and vitamins that should grab the bag of you and your baby you're going to miss out on so that's going to be one of your big problems and it can also upset your fertility for next time around and not many people realize that so your whole cycle can be pulled out and you might try have trouble falling pregnant again so there's some pretty serious side effects apart from the fetal alcohol syndrome that you might find affect you in your baby also too they found in some recent studies that the baby can be bored very restless and unsettled so even if it doesn't get fetal alcohol syndrome a lot of clinicians say they can see a baby that's been born to a mother who has been drinking during pregnancy there they actually unsettled hard hard to rest though they that they don't follow the normal patent of a newborn	"Thank you for your openness, Harriet. It sounds like you're aware that there might be potential issues, but you’re not quite sure what they are. That’s okay; this is a journey we can explore together.<br><br>What do you think could be some of the concerns related to drinking during pregnancy? Maybe there are things you've heard or thought about before? I’d love to hear what comes to mind for you."
Deepeval score = 0.7273	
okay so hopefully we can get that confidence up a bit and from what I hear you saying it sounds like you have been successful in the	It sounds like you see the importance of incorporating exercise into your life, especially given your diabetes, but you're

© 2026 Teferra BG et al. *JAMA Network Open*.

past making these changes related to lifestyle and incorporating exercise into your schedule is another really big change that might be really difficult for you to make	feeling quite overwhelmed and doubtful about your ability to make that change right now. A confidence level of two indicates that there are significant barriers you're facing. Can you tell me more about what specifically feels challenging when you think about starting to exercise?<br><br>It's understandable to feel that way with so much on your plate. You're clearly invested in your well-being, and recognizing the challenges is an important first step. What would it look like for you if you started making small changes toward exercising, even just a little?
Deepeval score = 0.7143	
okay so you realize that if you become more consistent with exercise and moving mores work you're going to feel better and you're held to be impacted positively	"That’s a significant realization, Brian, acknowledging that you're tired of the cycle of starting and stopping. It sounds like you’re really ready for more consistency, and that desire to lose a few pounds can be a strong motivator. It seems like you have some clear reasons for wanting to commit to this exercise routine.<br><br>I hear that you're feeling a sense of determination. On a scale of 1 to 10, with 10 being completely motivated, where do you think you fall right now in terms of motivation to stick with this plan? What makes you say that?"

© 2026 Teferra BG et al. *JAMA Network Open*.

eAppendix 4: Quadrant Plot

Quadrant-based comparison of cosine similarity and DeepEval scores. (A) Scatter plot of cosine similarity versus DeepEval score for all therapist–LLM response pairs. Dashed vertical and horizontal lines indicate median splits used to define four quadrants. Points are colored according to quadrant membership: both high, both low, cosine high/DeepEval low, and cosine low/DeepEval high.(B) Corresponding 2×2 heatmap showing the raw counts of dialogue turns falling into each quadrant defined in panel A.

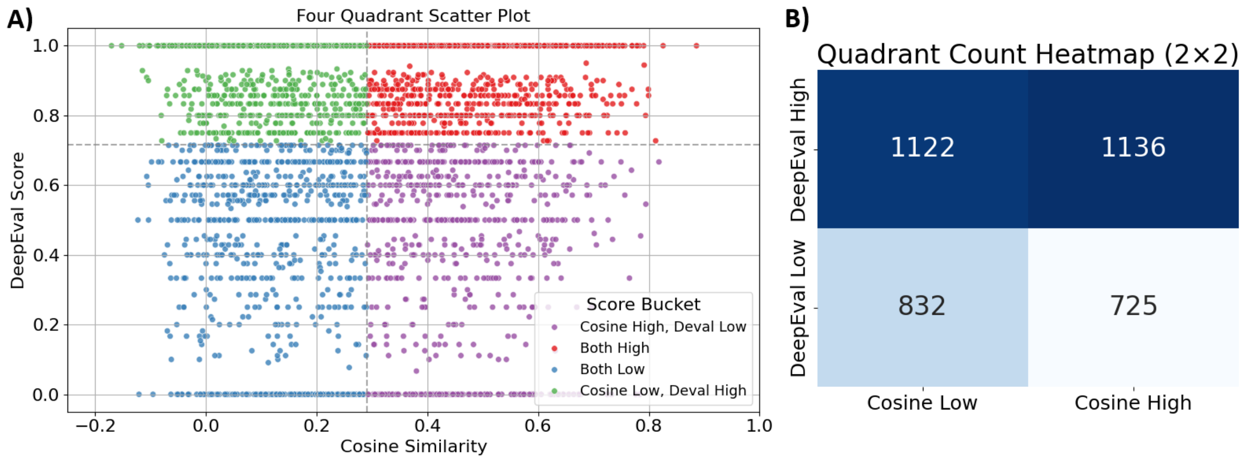

In the scatter plot, each point represents a single LLM-generated response compared with its corresponding human therapist response. Vertical and horizontal dashed lines denote the mean cosine similarity (0.29) and mean DeepEval score (0.72), dividing the space into four quadrants: both metrics high, both metrics low, high cosine similarity but low DeepEval score, and low cosine similarity but high DeepEval score. Colour coding highlights the distribution of points within each quadrant.

The accompanying heatmap summarizes the number of observations per quadrant, underscoring that cosine similarity and DeepEval capture distinct dimensions of alignment between LLM and therapist responses. This divergence supports the use of multi-metric evaluation when assessing LLM-generated dialogue in MI-coded therapeutic contexts.

© 2026 Teferra BG et al. *JAMA Network Open*.

eAppendix 5: Temporal Dynamics in Conversations

The histogram and Gaussian kernel density distribution of within-session similarity score slopes as conversation context accumulates is presented below. (A) Cosine similarity. (B) DeepEval.

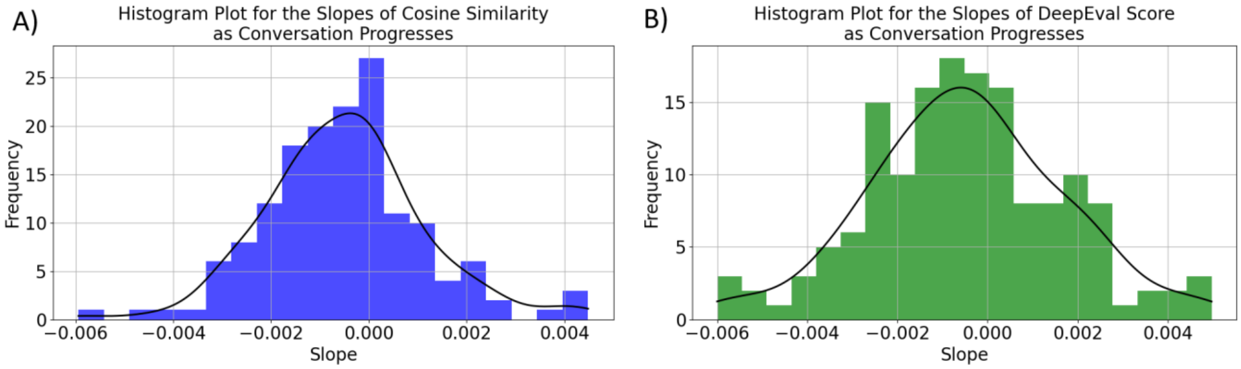

For each session, a linear slope was calculated to quantify whether similarity scores increased or decreased over the course of the dialogue. Most slopes cluster slightly below zero for both metrics, indicating a modest tendency for LLM–therapist alignment to decline as conversations advance. This downward trend may reflect challenges in long-range contextual tracking or growing conversational complexity over time. Gaussian kernel density curves (black lines) illustrate the distributional shape for each metric, showing consistent negative skew across both cosine similarity and DeepEval slopes.

© 2026 Teferra BG et al. *JAMA Network Open*.

eAppendix 6: Word Count Summary

Speaker	Mean Word Count	SD	Min	Max
**Therapist**	32·3	35·5	1	407
**LLM**	77·1	37·9	4	469

© 2026 Teferra BG et al. *JAMA Network Open*.

eAppendix 7: Error Cases Examples

1. Divergent Conversational Priorities:

The therapist focuses on verifying specific details, maintaining procedural accuracy, while the LLM transitions to a new topic, emphasizing continuity and client engagement.

Context
Therapist: I want you to have a seat here. To start off, I'm just going to verify your information real quick so we said your name's Larry seedorf is your date of birth let's see
Client: 129 68 okay

Response Comparison
Therapist: and it looks like are you still on same address as before
LLM: Great, thank you for confirming that, Mr. Seedorf. Now, let's talk about your medication. Are there any concerns or questions that you would have about it?

Cosine Similarity Score: 0.066
DeepEval Score: 0

2. Session Pacing Differences

It is near the end of the session, and the therapist is wrapping up the conversation while the LLM is still continuing with the conversation.

Context
Client: okay
Therapist: on Friday night
Client: okay

Response Comparison
Therapist: all right thanks
LLM: Fantastic. I can definitely hear how you've managed to utilize the stop and give techniques in your everyday life which is a true testament to your commitment to personal change. So, before we finish, what do you think are the things that could make it difficult to use these techniques when you interact with your mom the next time?

Cosine Similarity Score: -0.1209
DeepEval Score: 0

3. Contrasting Conversational Directions

The therapist is prompting for more information, while the LLM is trying to introduce new strategies

Context
Therapist: so you have a support group that gives you the ability to have fun and still drink
Client: yeah

© 2026 Teferra BG et al. *JAMA Network Open*.

Therapist: below risk levels
Client: yep

Response Comparison
Therapist: okay anything else
LLM: It sounds like you're recognizing some really important factors that support your ability to drink at low-risk levels. You mentioned the influence of your family history and how much you want to avoid that path, which shows a strong commitment to your health and well-being. That's significant. You also have a supportive group of friends who don't drink, which seems to create a positive environment for you.

Given this awareness and support, can you share what strategies you might use when faced with situations where drinking might be tempting? What do you think would help you maintain your goal of drinking below those risk levels?

Cosine Similarity Score: -0.0113
DeepEval Score: 0

4. Reflective Practices and Alignment

The therapist is confirming the patient's sentence, while the LLM tries to respond to what the client said with further MI strategies.

Context
Therapist: do you find it difficult to to get alcohol like you have to work to find it no
Client: no no

Response Comparison
Therapist Response: it's easy to find
LLM Response: It sounds like you had a challenging experience at the party, and it's understandable to feel a mix of emotions about it. It's great that your boss took it lightly, but I can see that you're still feeling some embarrassment.
You mentioned that you had intended to only have two drinks but ended up drinking more. Can you tell me more about that moment when you decided to have another drink? What were you thinking or feeling at that time?
Also, it's interesting to hear that you don't feel the need to cut down significantly on your drinking. It seems like you have a certain routine or social atmosphere where drinking is common. What do you think would help you feel more in control if you decided to cut back?
I want to affirm your honesty in sharing these experiences; it's not easy to talk about. If you were to think about a goal regarding your drinking, what might that look like for you?

Cosine Similarity Score: -0.0054
DeepEval Score: 0

© 2026 Teferra BG et al. *JAMA Network Open*.

5. Different Approaches to Follow-Up

The therapist focuses on verifying specific details, maintaining procedural accuracy, while the LLM transitions to a new topic, emphasizing continuity and client engagement.

Context
Therapist: so do you remember our conversation from our last visit that's when I give you the information about quitting smoking and the contact number for tobacco treatment specialist
Client: yes I remember but no I didn't quit and no I didn't call her

Response Comparison
Therapist Response: and why is that
LLM Response: I appreciate your honesty, Maria. It sounds like quitting smoking is still a challenge for you, and that's completely understandable. Change can be difficult, especially when it involves habits that have been a part of our lives for a long time.
Can you share with me what’s been holding you back from calling the specialist or making a change? What thoughts or feelings come up for you when you think about quitting?

Cosine Similarity Score: 0.0063
DeepEval Score: 0

© 2026 Teferra BG et al. *JAMA Network Open*.
