# Supplementary material for: Alignment of Large Language Model Responses With Human Therapists in Motivational Interviewing
Source: JAMA Netw Open. 2026 Mar 23;9(3):e262750. doi: 10.1001/jamanetworkopen.2026.2750 (PMC13010193; doi:10.1001/jamanetworkopen.2026.2750)
Supplement: Supplement 2. — Data Sharing Statement [file jamanetwopen-e262750-s002.pdf]

Data Sharing Statement

Teferra. Alignment of Large Language Model Responses With Human Therapists in Motivational Interviewing. *JAMA Netw Open*. Published March 23, 2026. doi:10.1001/jamanetworkopen.2026.2750

Data
Data available: Yes
Data types: Deidentified participant data
How to access data: The data used for this study is publicly available and can be found here: https://web.eecs.umich.edu/~mihalcea/downloads/HighLowQualityCounseling.zip
When available: With publication

Supporting Documents
Document types: Statistical/analytic code
How to access documents: The custom Python software developed for this study: implementation, metric computation, high/low consistency group assignment, statistical analyses and result visualizations is open-source and publicly available at https://github.com/teferrabg/TherapySimulation.git
When available: With publication

Additional Information
Who can access the data: The data is publicly available
Types of analyses: For any purpose
Mechanisms of data availability: Without investigator support
